# Supplementary material for: Ligand binding and conformational changes of SUR1 subunit in pancreatic ATP-sensitive potassium channels
Source: Protein Cell. 2018 Mar 28;9(6):553–67. doi: 10.1007/s13238-018-0530-y (PMC5966361; doi:10.1007/s13238-018-0530-y)
Supplement: Supplementary file 1 — Supplementary material 1 (PDF 1241 kb) [file 13238_2018_530_MOESM1_ESM.pdf]

Figure Supplement 1. Characterization of the K_{ATP} channel complex.
(A) Cartoon topology of SUR1-Kir6.2 fusion construct covalently linked by a flexible 39-residue linker (a dashed line). TMD, transmembrane domain; L0, loop 0; NBD, nucleotide-binding domain; OH, outer helix; PH, pore helix; IH, inner helix; βA, the first β strand of Kir6.2; IFH, interfacial helix; and CTD, cytoplasmic domain.
(B) Activation effect of Mg-ADP and NN414 (left panel), and inhibitory effect of GBM and ATP (right panel) on the macroscopic currents of SUR1-Kir6.2 fusion construct in an inside-out mode.
(C) Size exclusion chromatography (SEC) elution profiles of SUR1-Kir6.2 K_{ATP} fusion protein. Pooled fractions between dashed lines were used for cryo-EM sample preparation.
(D) SDS-PAGE of purified K_{ATP} fusion protein corresponding to the indicated SEC fractions. Fractions labeled with stars were pooled and concentrated for cryo-EM grids preparation.

Figure Supplement 2. Workflow for cryo-EM data processing of K_{ATP} in complex with ATPγS and GBM (ATP + GBM state).
(A) Representative raw micrograph.
(B) Representative two-dimensional class averages of the K_{ATP} channel.
(C) Flow-chart of EM data processing. Focused classification on Kir6.2 CTD and SUR1 NBD2, and focused refinement on SUR1 ABC transporter module were applied to improve the map quality of corresponding regions. Masked and corrected gold-standard Fourier shell correlation (FSC) curves of the final refinement are shown below the maps after post-processing. Resolution estimation was based on the criterion of FSC 0.143 cutoff.

Figure Supplement 3. Structure of K_{ATP} in complex with ATPγS (ATP state).
(A) Workflow for cryo-EM data processing of K_{ATP} in ATP state. Flow-chart of 3D classification, refinement, and focused 3D classification on Kir6.2 CTD and subsequent refinement for "T state" and "R state". Masked and corrected gold-standard Fourier shell correlation (FSC) curves of the final refinement are shown below the maps after post-processing. Resolution estimation was based on the criterion of FSC 0.143 cutoff.
(B) Top view of structural comparison of "T state" (left) and "R state" (right) structures between the ATP (green) and ATP + GBM (gray) states.

Figure Supplement 4. Cryo-EM map of K_{ATP} in the ATP + GBM state and the GBM binding site.
(A) Local EM densities of ATPγS in "T state" (top) and "R state" (bottom) structures of the ATP + GBM state. Maps are colored the same as in Figure 1A.
(B) Cryo-EM map around the lasso motif in the ATP + GBM (top) and Mg-ADP states (bottom, described later). Extra density with unknown identity is colored in green.
(C) Different conformation of glibenclamide bound in K_{ATP} (top) and human AKR1C (bottom) (PDB: 4YVP).
(D) Cryo-EM density of GBM.
(E) Putative glibenclamide interactions with SUR1. Blue ovals represent residues on TMD1 and magenta ovals represent residues on TMD2.

(F) Superposition of SUR1 ABC transporter module structures in the ATP + GBM state solved using different constructs. Structure of SUR1-Kir6.2 fusion construct in this study is shown in grey. Structure of SUR1+Kir6.2 as separated peptides (PDB: 6BAA) is colored. Transmembrane helices from the first half of TMD of SUR1 ABC transporter module are aligned. Angles between helices M10 and M16 in the two structures are shown below.

Figure Supplement 5. Workflow for cryo-EM data processing of K_{ATP} in the Mg-ADP state.
Flow-chart of 3D classification and subsequent refinement. To improve the map quality certain regions, focused classification and refinement were employed similarly as in Extended Data Fig. 2c. Masked and corrected gold-standard Fourier shell correlation (FSC) curves of the final refinement are shown below the maps after post-processing. Resolution estimation was based on the criterion of FSC 0.143 cutoff.

Figure Supplement 6. Structural comparison of K_{ATP} in different states.
(A) Top view of structural comparison between the Mg-ADP (“T state”) and (“propeller form”, PDB: 6C3P) states by aligning Kir6.2.
(B) Superposition of the ABC module between the Mg-ADP (colored) and Mg-ATP&ADP (grey) states.
(C) Local EM density maps of inhibitory site bound with ADP (top), ATPγS (middle), and ATP (EMD-7073 and PDB: 6BAA) (bottom) in different states. Maps of Kir6.2 are colored in green. Nucleotide densities are show as blue meshes with nucleotide model shown as sticks.
(D) Similar asymmetric NBD dimer induced by the Mg-ADP (colored spheres) or Mg-ATP/Mg-ADP (gray spheres) molecules.

Figure Supplement 7. Extra densities on the map of SUR1 in the Mg-ADP state.
(A) Chemical structure of NN414 (6-chloro-3-[[1-methylcyclopropyl]amino]-4H-thieno[3,2-e]-1,2,4-thiadiazine 1,1-dioxide tifenazoxide).
(B) Local EM density maps surrounded by M10-12 and M17 in the Mg-ADP state (top) with one extra density (density 1: yellow). The same site in the Mg-ATP&ADP state (bottom, EMD-7338, PDB: 6C3O) is apo.
(C) Local EM density maps surrounded by M8 and M15–17 in the Mg-ADP state (top) the other extra density (density 2: red). The same site in the Mg-ATP&ADP state EMD-7338, PDB: 6C3O) is apo. Figures are created from 4.2 Å, focused refinement map of SUR1 ABC transporter module. The cryo-EM density maps are contoured to the same level as in panel (B). Densities of SUR1 molecule are colored the same as in Figure 1.
(D) Superposition of the structures with (Mg-ADP state) and without NN414 (Mg-ATP&ADP state) by aligning the TMD domain of SUR1. Local conformational changes of transmembrane helices (M9-11) at density 1 are indicated by red arrows. A surface representation of density 1 is shown in yellow.

Figure S1

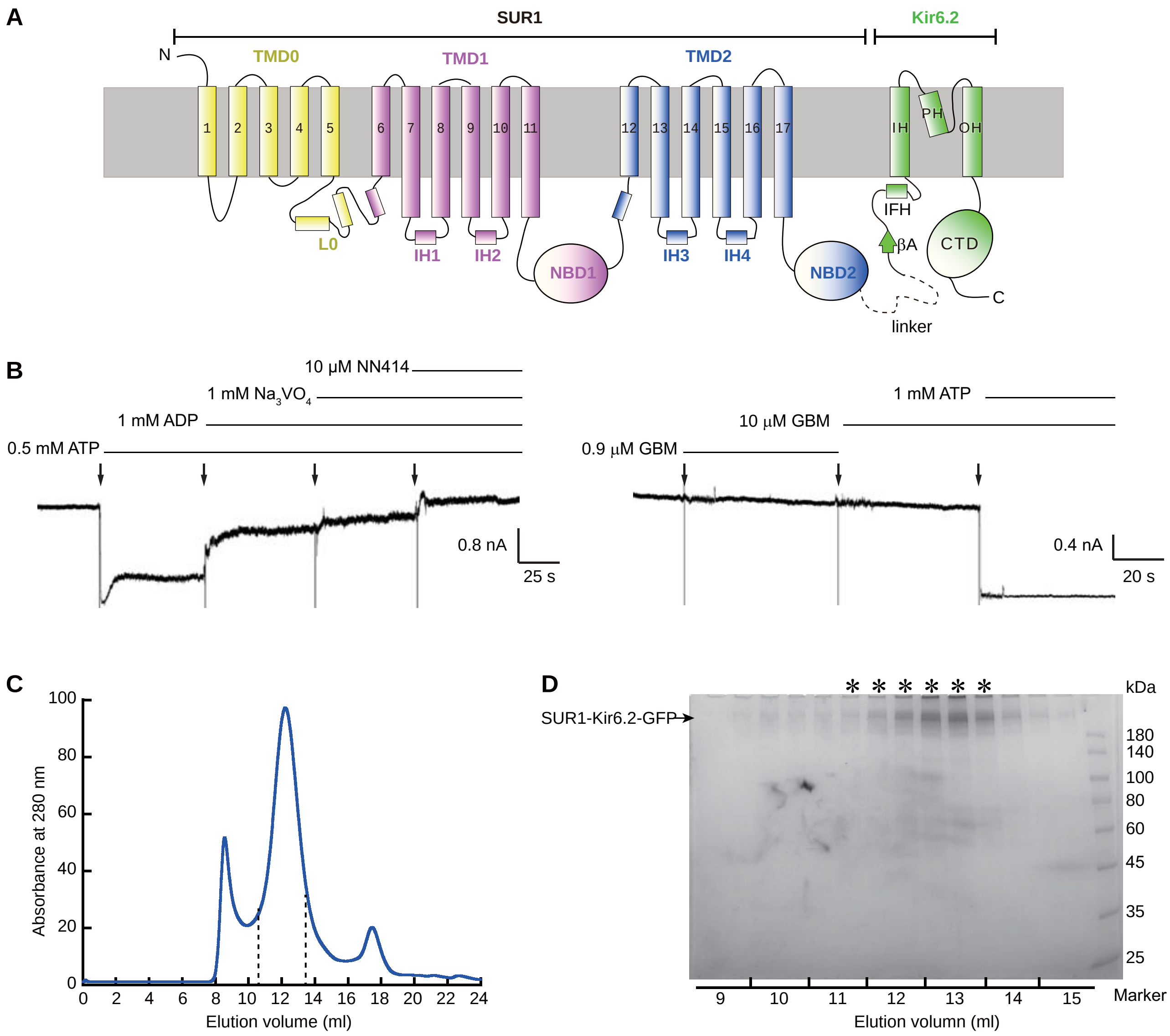
A
SUR1
Kir6.2
N
TMD0
TMD1
TMD2
1
2
3
4
5
6
7
8
9
10
11
12
13
14
15
16
17
IH
PH
OH
L0
IH1
IH2
NBD1
IH3
IH4
NBD2
IFH
βA
CTD
C
linker
B
10 μM NN414
1 mM Na$_3$VO$_4$
1 mM ADP
0.5 mM ATP
0.8 nA
25 s
1 mM ATP
10 μM GBM
0.9 μM GBM
0.4 nA
20 s
C
Absorbance at 280 nm
Elution volume (ml)
D
SUR1-Kir6.2-GFP
kDa
180
140
100
80
60
45
35
25
9
10
11
12
13
14
15
Marker
Elution volumn (ml)

Figure S2

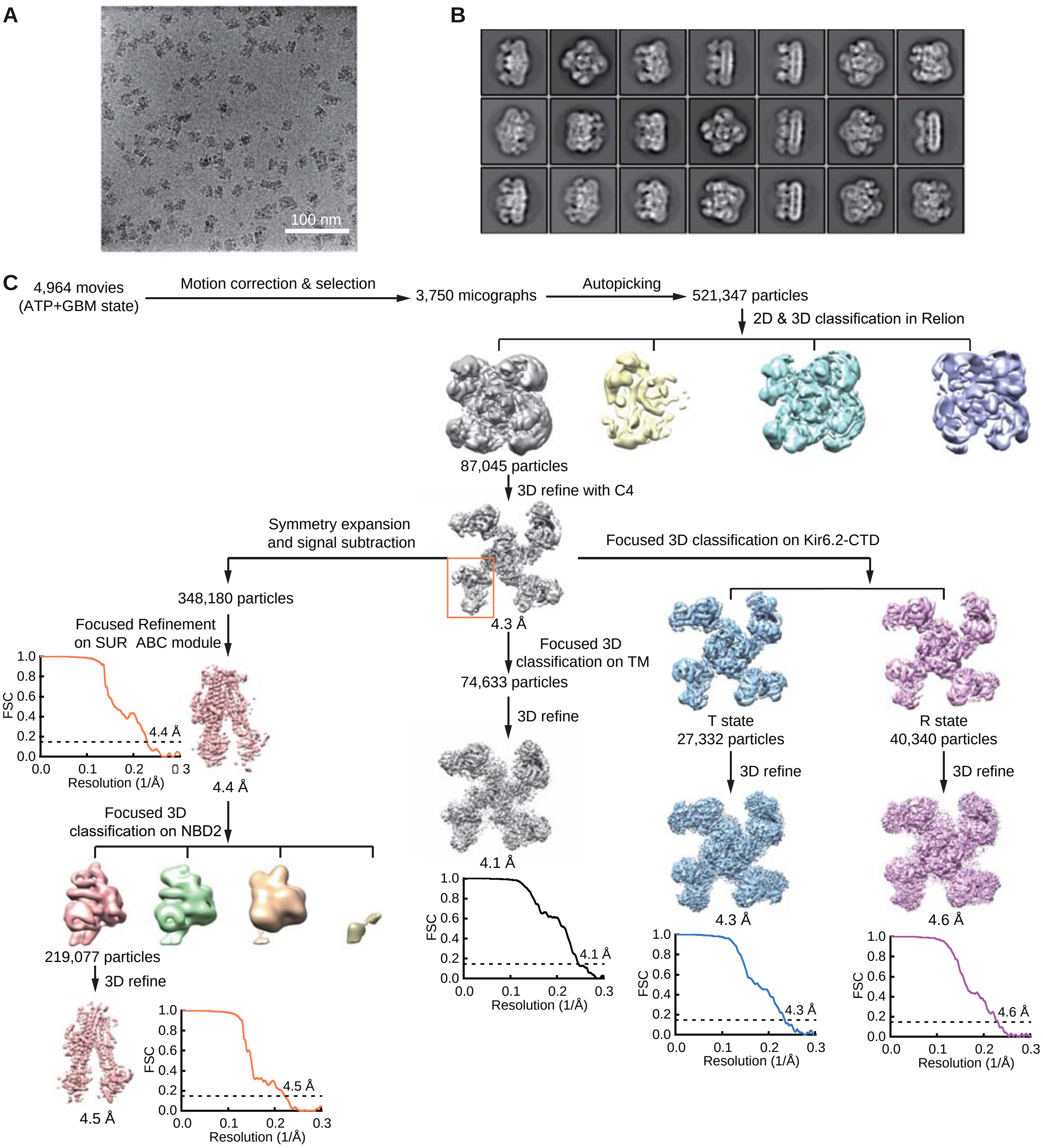

A
B
100 nm
C
4,964 movies
(ATP+GBM state)
Motion correction & selection
3,750 micographs
Autopicking
521,347 particles
2D & 3D classification in Relion
87,045 particles
3D refine with C4
Symmetry expansion
and signal subtraction
Focused 3D classification on Kir6.2-CTD
348,180 particles
4.3 Å
Focused Refinement
on SUR ABC module
Focused 3D
classification on TM
74,633 particles
3D refine
T state
27,332 particles
R state
40,340 particles
3D refine
3D refine
4.4 Å
4.1 Å
4.3 Å
4.6 Å
Focused 3D
classification on NBD2
219,077 particles
3D refine
4.5 Å
FSC
Resolution (1/Å)

Figure S3

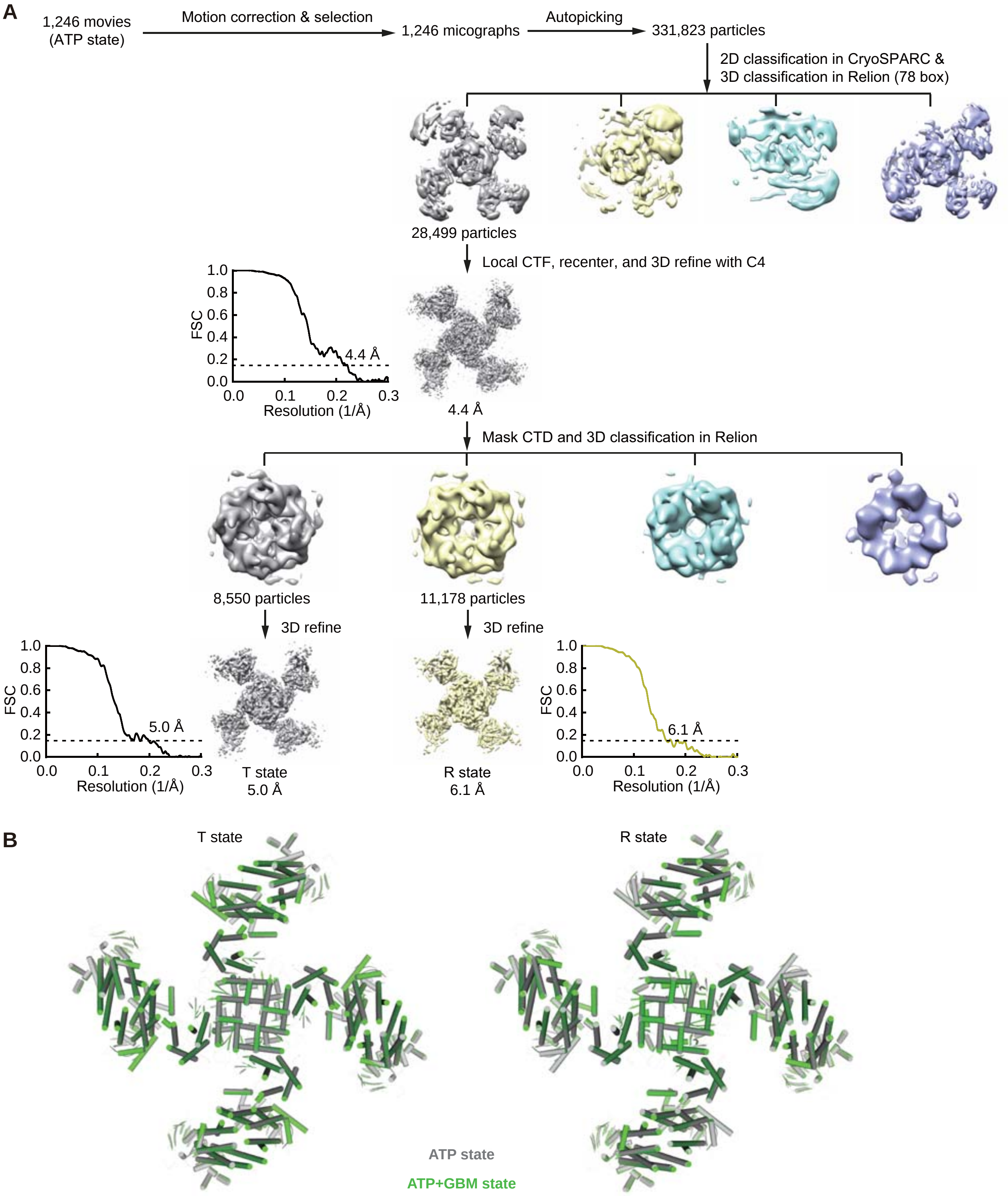
A
1,246 movies
(ATP state)
Motion correction & selection
1,246 micrographs
Autopicking
331,823 particles
2D classification in CryoSPARC &
3D classification in Relion (78 box)
28,499 particles
Local CTF, recenter, and 3D refine with C4
FSC
Resolution (1/Å)
4.4 Å
4.4 Å
Mask CTD and 3D classification in Relion
8,550 particles
11,178 particles
3D refine
3D refine
5.0 Å
T state
5.0 Å
R state
6.1 Å
6.1 Å
B
T state
R state
ATP state
ATP+GBM state

Figure S4

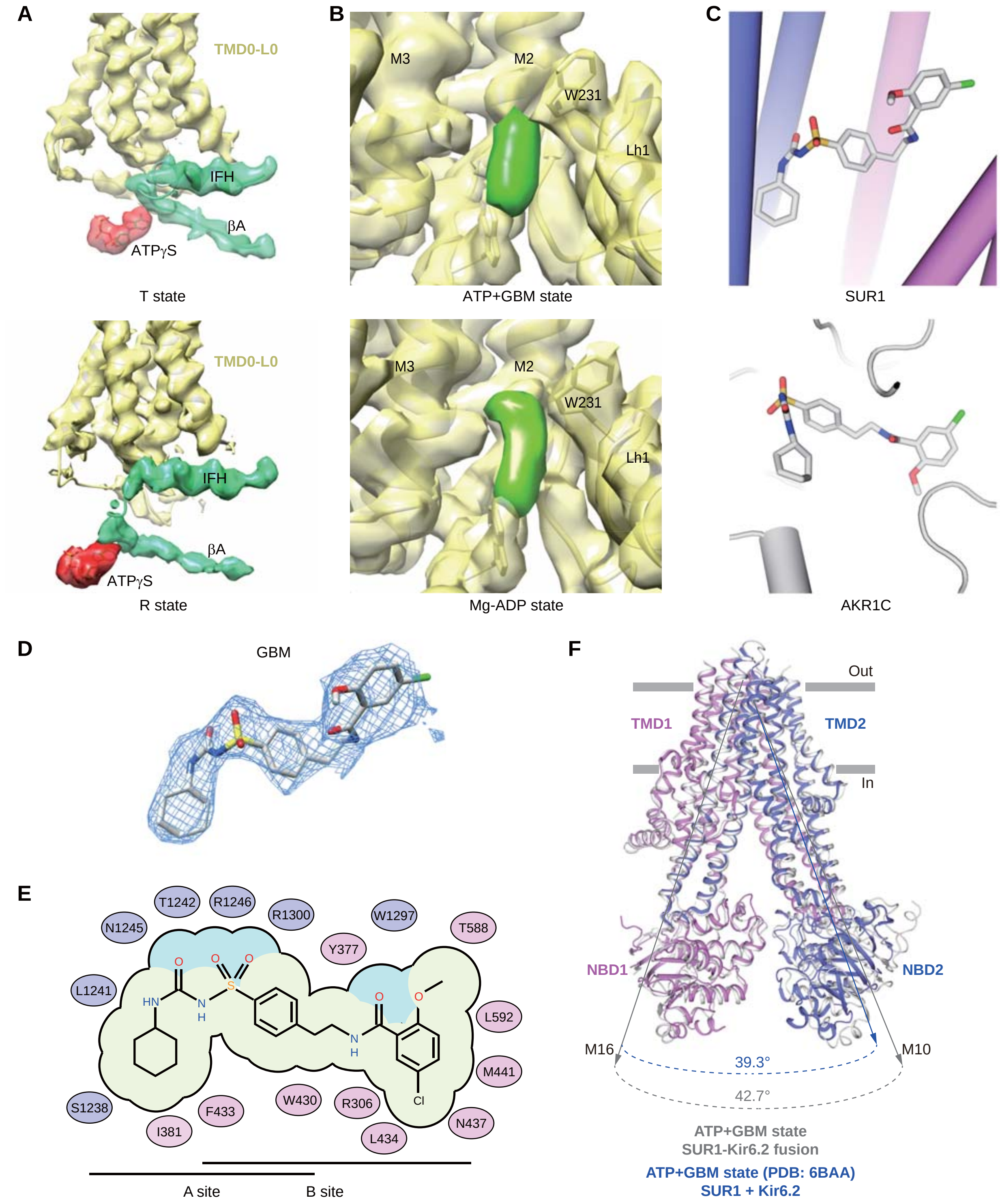

A
TMD0-L0
IFH
βA
ATPγS
T state
TMD0-L0
IFH
βA
ATPγS
R state
B
M3
M2
W231
Lh1
ATP+GBM state
M3
M2
W231
Lh1
Mg-ADP state
C
SUR1
AKR1C
D
GBM
E
T1242
R1246
N1245
R1300
W1297
T588
Y377
L1241
L592
M441
S1238
F433
W430
R306
N437
I381
L434
HN
N
H
S
O
O
O
N
H
O
O
Cl
A site
B site
F
Out
TMD1
TMD2
In
NBD1
NBD2
M16
M10
39.3°
42.7°
ATP+GBM state
SUR1-Kir6.2 fusion
ATP+GBM state (PDB: 6BAA)
SUR1 + Kir6.2

Figure S5

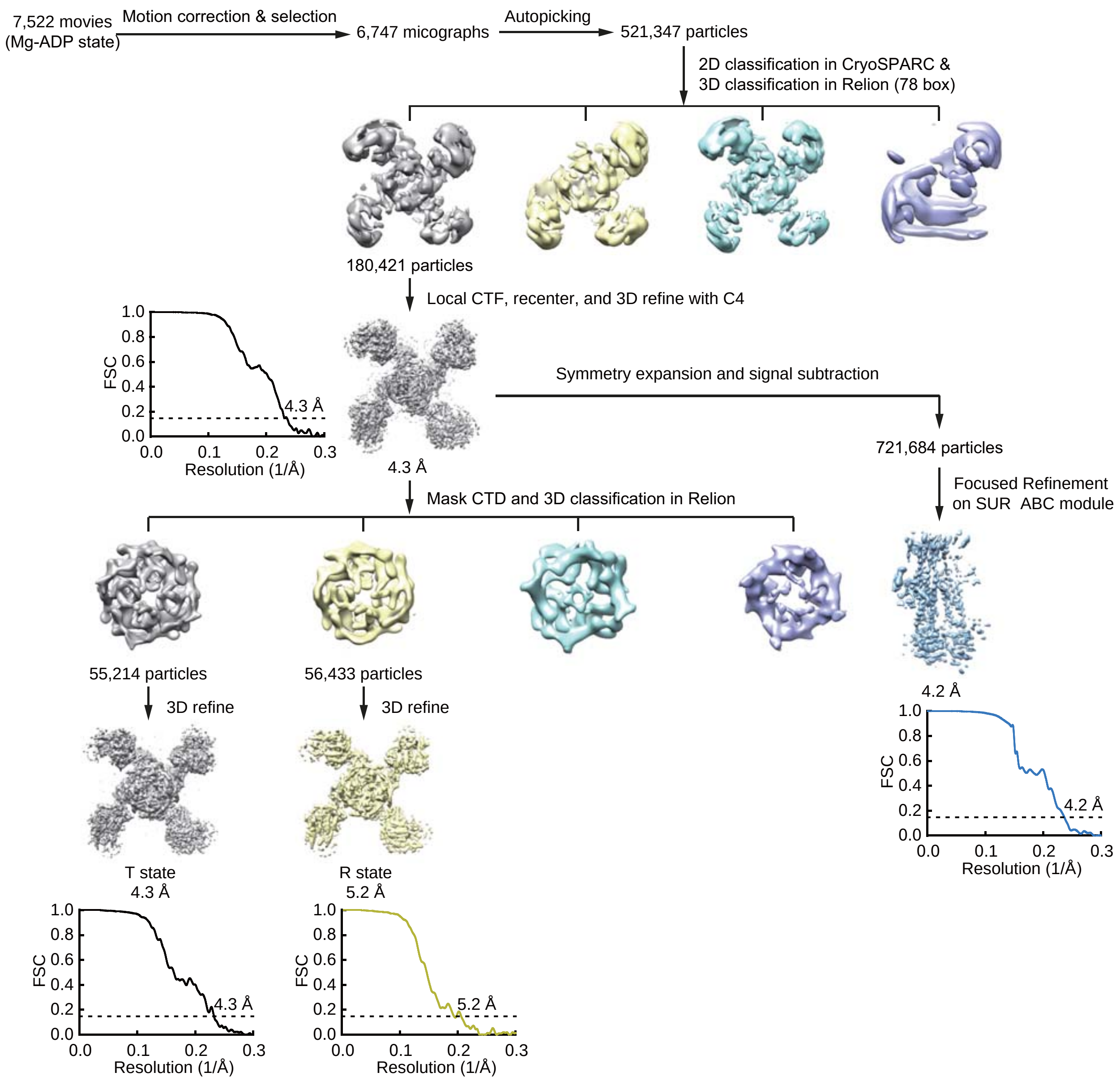
7,522 movies
(Mg-ADP state)
Motion correction & selection
6,747 micrographs
Autopicking
521,347 particles
2D classification in CryoSPARC &
3D classification in Relion (78 box)
180,421 particles
Local CTF, recenter, and 3D refine with C4
FSC
Resolution (1/Å)
4.3 Å
Symmetry expansion and signal subtraction
721,684 particles
Focused Refinement
on SUR ABC module
4.2 Å
Mask CTD and 3D classification in Relion
55,214 particles
56,433 particles
3D refine
3D refine
T state
4.3 Å
R state
5.2 Å

Figure S6

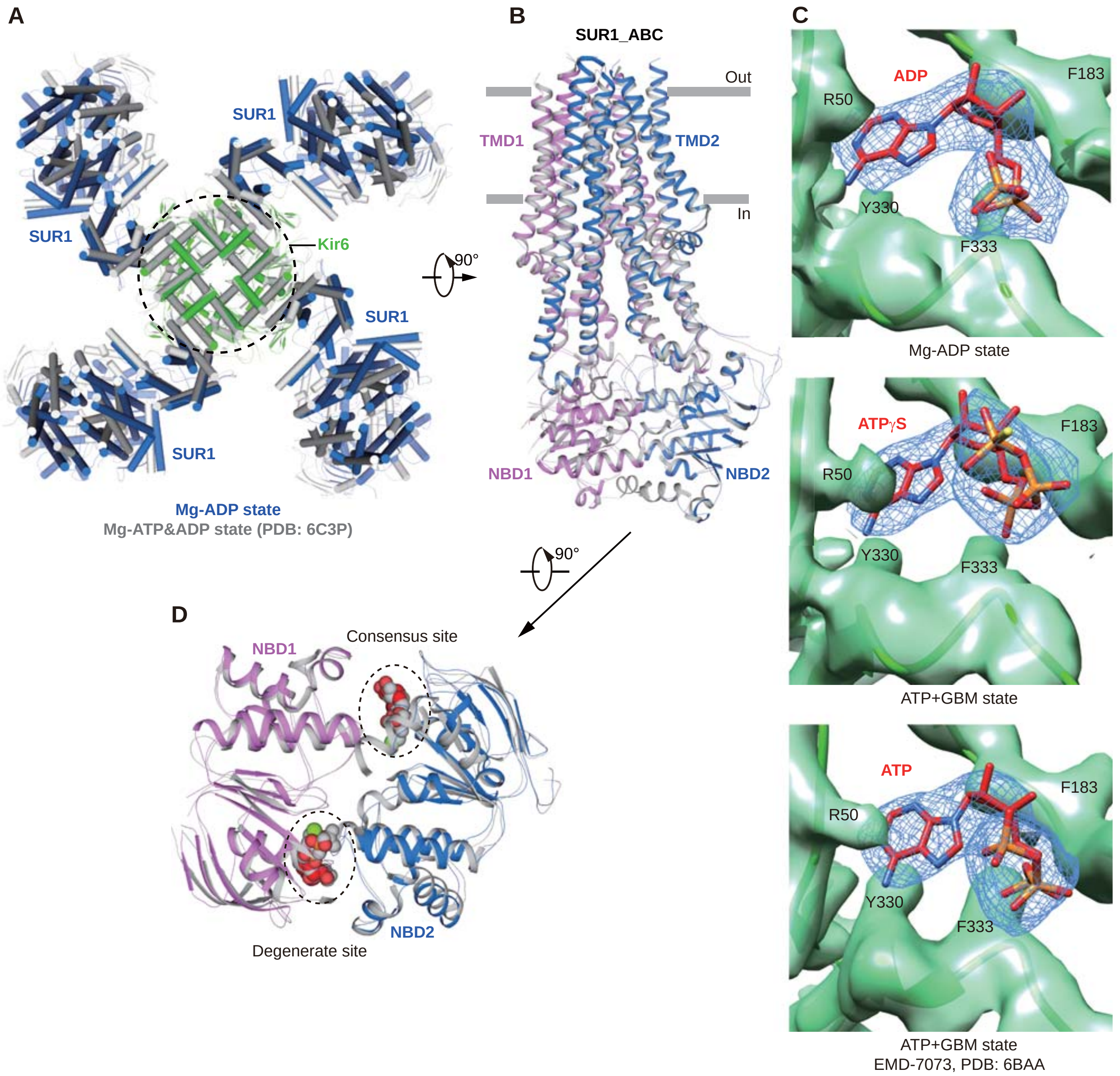
A
SUR1
SUR1
Kir6
SUR1
SUR1
Mg-ADP state
Mg-ATP&ADP state (PDB: 6C3P)
90°
B
SUR1_ABC
Out
TMD1
TMD2
In
NBD1
NBD2
90°
C
ADP
R50
F183
Y330
F333
Mg-ADP state
ATPγS
F183
R50
Y330
F333
ATP+GBM state
ATP
F183
R50
Y330
F333
ATP+GBM state
EMD-7073, PDB: 6BAA
D
NBD1
Consensus site
NBD2
Degenerate site

Figure S7

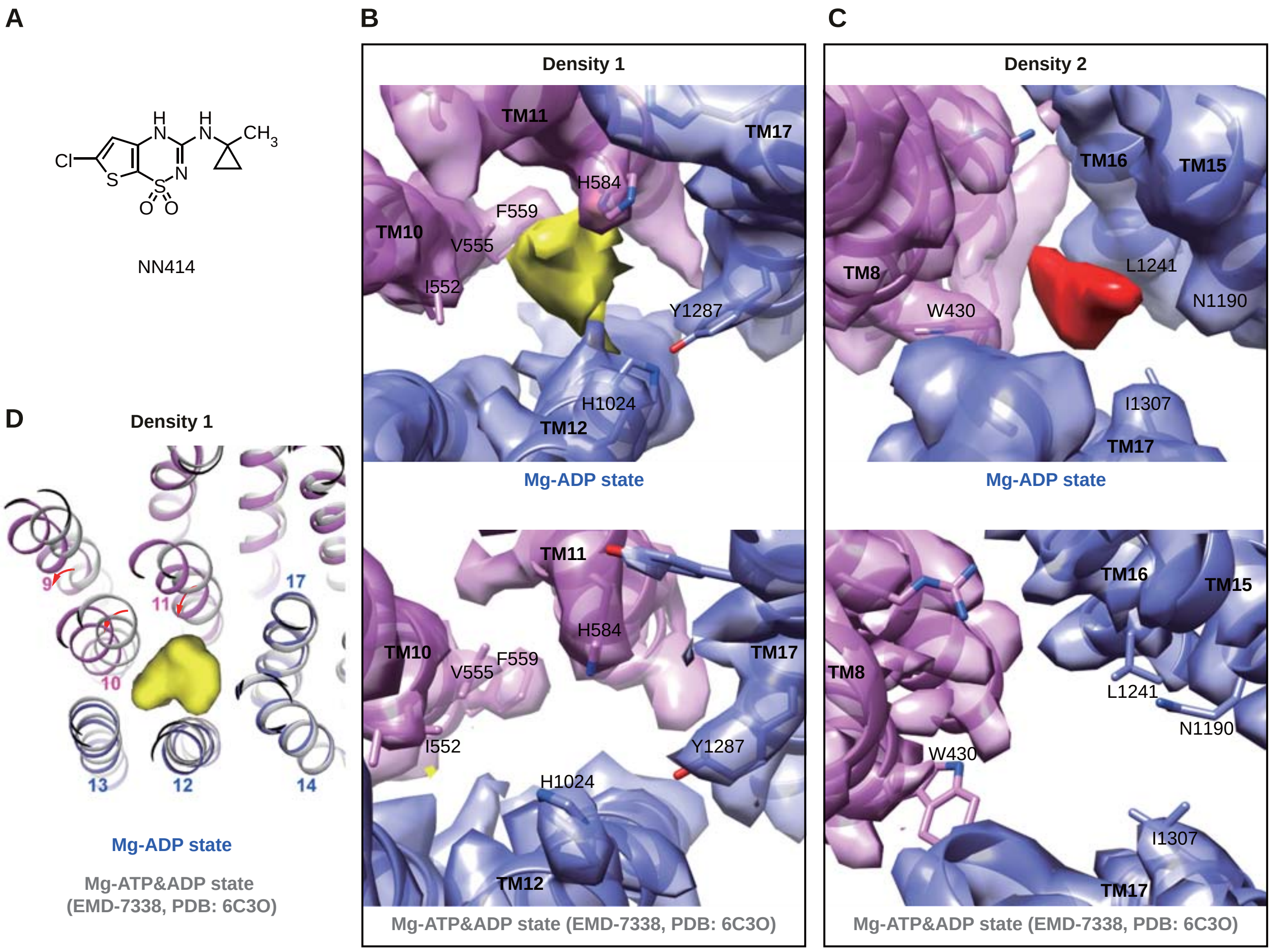
A
B
C
Density 1
Density 2
Cl
S
N
H
CH3
O
NN414
TM11
TM17
H584
F559
TM10
V555
I552
Y1287
H1024
TM12
TM16
TM15
TM8
L1241
N1190
W430
I1307
Mg-ADP state
Mg-ATP&ADP state (EMD-7338, PDB: 6C3O)
D
9
11
10
17
13
12
14
Mg-ATP&ADP state
(EMD-7338, PDB: 6C3O)

Table S1.
Cryo-EM data collection, refinement and validation statistics of the ATP+GBM state of K_{ATP}.

	Focus on TM<br>EMD-6831<br>5YKE	Class 1 of Kir6.2 CTD<br>EMD-6832<br>5YKF	Class 2 of Kir6.2 CTD<br>EMD-6833<br>5YKG	Focus on SUR1 ABC module<br>EMD-6847<br>5YW7	Class 1 of SUR1 NBD2
**Data collection and processing**					
Magnification			47,400×		
Voltage (kV)			300		
Electron exposure ($e^{-}/Å^2$)			52		
Defocus range (μm)			-1.5 to -3.5		
Pixel size (Å)			1.055		
Symmetry imposed	*C4*	*C4*	*C4*	*C1*	*C1*
Final particle images (no.)	74,633	27,332	40,340	348,180	219,077
Map resolution (Å)	4.1	4.3	4.6	4.4	4.5
FSC threshold	0.143	0.143	0.143	0.143	0.143
Map sharpening *B* factor ($Å^2$)	-204	-187	-210	-300	-307
**Refinement**					
Model composition					
Non-hydrogen atoms	30,636	50,988	50,868	8,944	
B factors ($Å^2$)					
Non-hydrogen atoms	130.49	151.43	152.64	168.47	
R.m.s. deviations					
Bond lengths (Å)	0.005	0.005	0.003	0.003	
Bond angles (°)	0.780	0.765	0.654	0.658	
Validation					
MolProbity score	2.52	2.71	2.61	2.57	
Clashscore	11.31	15.14	13.19	13.99	
Poor rotamers (%)	7.60	8.16	7.90	6.49	
Ramachandran plot					
Favored (%)	95.93	95.15	95.65	95.72	
Allowed (%)	3.65	4.42	4.23	4.28	
Disallowed (%)	0.42	0.44	0.12	0.00	

Table S2.
Cryo-EM data collection, refinement and validation statistics of the ATP state of K_{ATP}.

	All particles<br>EMD-6848<br>5YW8	Class 1 of Kir6.2 CTD<br>EMD-6849<br>5YW9	Class 2 of Kir6.2 CTD<br>EMD-6850<br>5YWA
**Data collection and processing**			
Magnification		37,300×	
Voltage (kV)		300	
Electron exposure ($e^-/Å^2$)		52	
Defocus range (μm)		-1.5 to -3.5	
Pixel size (Å)		1.34	
Symmetry imposed		*C4*	
Final particle images (no.)	28,499	8,550	11,178
Map resolution (Å)	4.4	5.0	6.1
FSC threshold	0.143	0.143	0.143
Map sharpening *B* factor ($Å^2$)	-198	-161	-195
**Refinement**			
Model composition			
Non-hydrogen atoms	50,524	50,524	50,496
B factors ($Å^2$)			
Non-hydrogen atoms	219.24	219.24	505.25
R.m.s. deviations			
Bond lengths (Å)	0.005	0.005	0.004
Bond angles (°)	0.796	0.796	0.693
Validation			
MolProbity score	2.81	2.81	2.72
Clashscore	19.01	19.00	17.20
Poor rotamers (%)	8.11	8.11	7.91
Ramachandran plot			
Favored (%)	95.13	95.13	95.65
Allowed (%)	4.43	4.43	4.17
Disallowed (%)	0.44	0.44	0.19

Table S3.
Cryo-EM data collection, refinement and validation statistics of the Mg-ADP state of K_{ATP}.

	All particles	Class 1 of Kir6.2 CTD EMD-6852 5YWC	Class 2 of Kir6.2 CTD EMD-6851 5YWB	Focus on SUR1 ABC module EMD-6853 5YWD
**Data collection and processing**				
Magnification		47,400×		
Voltage (kV)		300		
Electron exposure ($e^{-}/Å^2$)		52		
Defocus range (μm)		-1.5 to -3.5		
Pixel size (Å)		1.055		
Symmetry imposed	*C4*	*C4*	*C4*	*C1*
Final particle images (no.)	180,421	55,214	56,433	721,684
Map resolution (Å)	4.3	4.3	5.2	4.2
FSC threshold	0.143	0.143	0.143	0.143
Map sharpening *B* factor ($Å^2$)	-227	-188	-265	-269
**Refinement**				
Model composition				
Non-hydrogen atoms		51,108	50,928	8,997
B factors ($Å^2$)				
Non-hydrogen atoms		283.99	300.02	203.33
R.m.s. deviations				
Bond lengths (Å)		0.004	0.004	0.003
Bond angles (°)		0.796	0.776	0.718
Validation				
MolProbity score		2.69	2.62	2.49
Clashscore		14.70	13.27	12.19
Poor rotamers (%)		7.97	7.66	6.20
Ramachandran plot				
Favored (%)		95.26	95.50	95.87
Allowed (%)		4.47	4.07	4.13
Disallowed (%)		0.37	0.43	0.00
